# Hydroxyapatite and Fluorapatite in Conservative Dentistry and Oral Implantology—A Review

**DOI:** 10.3390/ma12172683

**Published:** 2019-08-22

**Authors:** Kamil Pajor, Lukasz Pajchel, Joanna Kolmas

**Affiliations:** Analytical Group, Department of Analytical Chemistry and Biomaterials, Faculty of Pharmacy with Laboratory Medicine Division, Medical University of Warsaw, 02-097 Warsaw, Poland

**Keywords:** hydroxyapatite, fluorapatite, dentistry, calcium phosphates

## Abstract

Calcium phosphate, due to its similarity to the inorganic fraction of mineralized tissues, has played a key role in many areas of medicine, in particular, regenerative medicine and orthopedics. It has also found application in conservative dentistry and dental surgery, in particular, as components of toothpaste and mouth rinse, coatings of dental implants, cements, and bone substitute materials for the restoration of cavities in maxillofacial surgery. In dental applications, the most important role is played by hydroxyapatite and fluorapatite, i.e., calcium phosphates characterized by the highest chemical stability and very low solubility. This paper presents the role of both apatites in dentistry and a review of recent achievements in the field of the application of these materials.

## 1. Introduction

In recent decades, one has been able to observe huge progress in the field of dentistry. This results not only from the development of dental techniques and methods of therapy but also from significant developments in biomaterial engineering. The science of biomaterials is constantly increasing due to innovative modifications of already known materials or completely new biomaterials for applications in dentistry. Biodegradable polymers, bioactive ceramics, bioglass or metals covered with a layer of material facilitating osseointegration and, above all, composite materials are the main directions in the development of dental biomaterials [1,2,3,4].

One of the more widely studied groups of materials comprises inorganic calcium phosphates (CaPs). It is worth noting that synthetic calcium phosphate material was first used to repair and regenerate bone tissue in 1920 by Albee, who employed crystalline calcium phosphate to repair surgically induced bone defects in rabbits [5,6,7]. Bioceramics based on calcium phosphate began to enjoy greater interest only at the end of the 1960s, when they were seen as biomaterials that could induce the reconstruction and repair of bone defects [8]. In the 1970s, researchers began to pay attention to the use of calcium phosphates in dental surgery. Particularly noteworthy is the work of Denissen and de Groot, in which cylinders made of hydroxyapatite (HA) were used as dental implants. In the 1980s, synthetic calcium phosphates were permanently introduced in implantology, and the research on the use of natural CaPs accelerated [7]. Currently, the possibility of using calcium phosphates as carriers of biologically active substances is being sought [7,9,10,11].

The reason why CaPs are such a popular object of research and applications is their similarity to the inorganic fraction of mineralized tissues, i.e., bone, enamel, dentin and cementum [7,12,13].

### 1.1. Dental Mineralized Tissues

**Figure 1:**
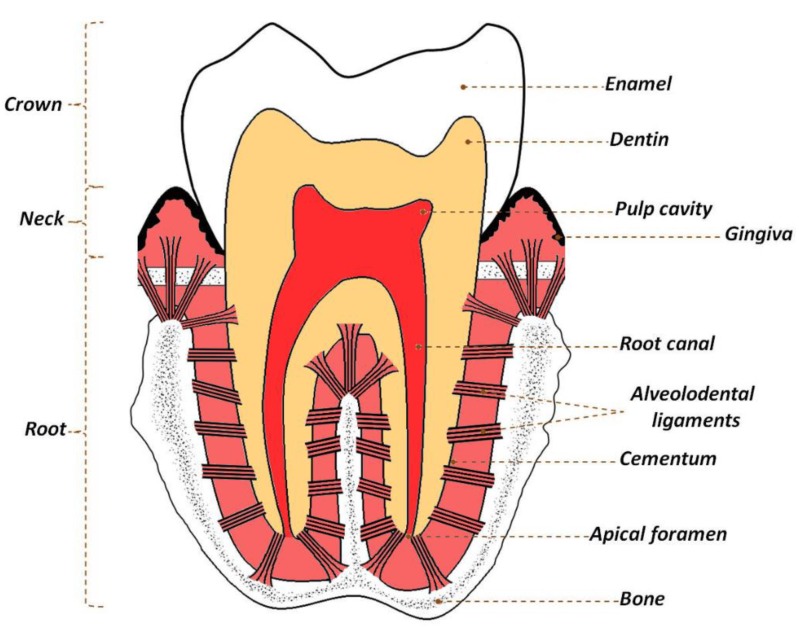
Schematic picture of a molar tooth.

Enamel is the tissue that covers the crown of the tooth (Figure 1). It consists of thin, corrugated and very elongated structural subunits which run through the entire thickness of the enamel-these are called enamel prisms and are of an inter-prismatic substance. The tooth enamel mainly consists of inorganic substance (98 wt.%) [8,14,15]. The prisms are made of very large crystals of biological apatite, the size of which significantly exceeds that found in other mineralized tissues (their length can reach up to 100 μm, with only a few dozen nm in terms of width and thickness) and their number varies between five and 12 million prisms on one tooth crown [14,16,17,18].

The neck (dental cervix) and root of the tooth are covered with a thin layer of cementum (cement), resembling bone tissue in the structure and chemical composition. The intercellular substance in the dental cement consists of collagen (mainly types I and III) and other non-collagenic proteins, e.g., fibronectin or vitronectin. The inorganic fraction of cement (65 wt.%), as in enamel, is mainly a biological apatite, which is characterized by a much lower degree of crystallinity than enamel apatite. This makes it more susceptible to dissolution, but also to the adsorption of “foreign” ions on the surface [8,14,16,17,18].

Below the enamel and cement, there is dentin, which in the majority (72 wt.%) is composed of inorganic matter, mainly apatite, organic components and water. Dentin is the main building material of the tooth, forming a part of the crown and the root of the tooth, giving it its basic shape. The main component of the organic matrix is collagen (92 wt.%), appearing in the form of thin fibers. The rest are non-collagen proteins, proteoglycans, growth factors, phospholipids and enzymes [15,16,17,18]. Noteworthy are some of the non-collagen proteins (phosphophoryn and dentin sialoprotein) and phospholipids, which play an important role in the development of the tooth (dentin mineralization). As mentioned above, inorganic matter accounts for 72% of dentin mass, but 50% of its volume, that makes it a tissue harder than bone. Apatite crystals that build this tissue are larger than those found in bone, but much smaller than those in enamel. Newly formed dentin apatite crystals are arranged in spherical regions called calcospherites [16,17,18].

Enamel, dentin and cementum apatite (i.e., generally biological apatite) has a complex composition. Generally, it is a carbonated HA with a reduced calcium content and hydroxyl groups in relation to the stoichiometric HA, with the general formula Ca_10_(PO_4_)_6_(OH)_2_ [19]. In addition, biological apatite is characterized by the presence of many other ions, e.g., Mg^2+^, K^+^, Na^+^, Zn^2+^, Mn^2+^, SiO_4_^4−^ and Cl^−^, partially substituted in the crystal structure (instead of calcium ions, phosphate ions or hydroxyl ions) and partially located on the surface of crystals, in the so-called hydrated surface layer [7,8,20]. It is worth noting that the partial replacement of hydroxyl ions –OH in the enamel apatite structure by fluoride ions leads to increased hardness and also the stability of crystals, as well as protecting the enamel against the effect of too low a pH in the oral cavity [8,21,22,23,24]. In general, it is important to note that the composition of biological apatite is not constant and depends on many factors: The type of tissue, the age, health and diet of the individual, environmental factors, etc.

**Figure 2:**
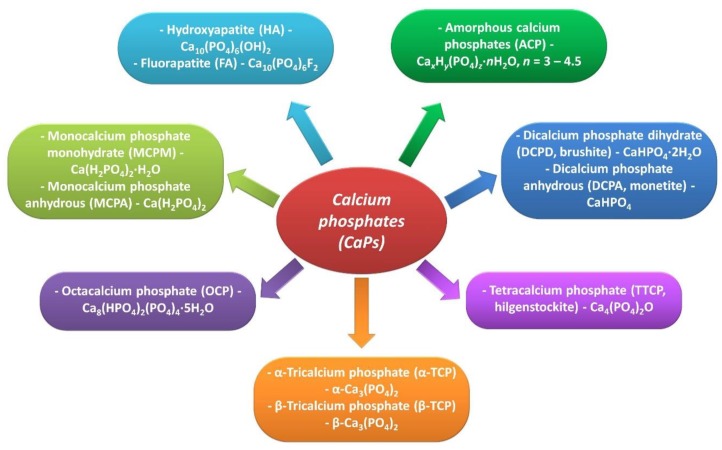
Calcium phosphates in biomedical applications.

Synthetic calcium phosphates represent a fairly large group of amorphous and crystalline compounds (Figure 2).

Some of them play a minor role in biomedicine (e.g., monocalcium phosphate monohydrate- MCPM and monocalcium phosphate anhydrous-MCPA), mainly due to very good solubility in water and a low Ca/P ratio [25]. Among the calcium phosphates that have found application in dentistry, we can mention above all HA and fluorapatite (FA). As these materials are characterized by high bioactivity and biocompatibility as well as the lack of toxic and allergenic properties, they are used as coatings for metallic implants, components of composite materials, bone substitute materials, bone cements, components of dental materials and toothpastes [7,8,26].

The aim of this work is to present the current state of knowledge and progress in the development and the applicability of materials containing HA and FA in conservative dentistry and dental surgery.

### 1.2. Hydroxyapatite and Fluorapatite

**Figure 3:**
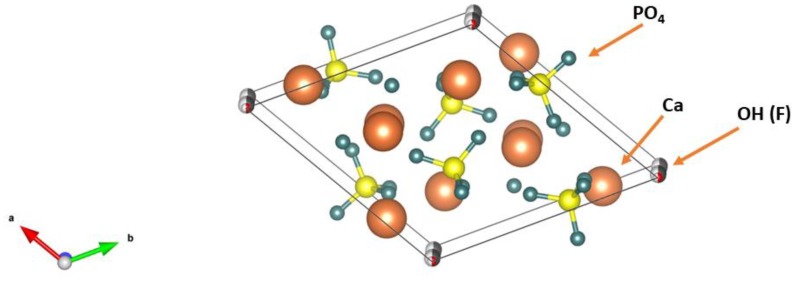
Schematic pictures of unit cells for: (a) HA and (b) FA.

HA and FA belong to a large group of calcium apatites with the formula Ca_10_(PO_4_)_6_X_2_. If X describes hydroxyl groups, the mineral is called HA (Ca_10_(PO_4_)_6_(OH)_2_, and, in the case of fluoride ions, FA Ca_10_(PO_4_)_6_F_2_. The mineral containing both fluoride and hydroxyl ions is called hydroxyfluorapatite (HFA). In turn, the structure of such a mineral will be described by the formula Ca_10_(PO_4_)_6_(OH_x_F_y_) [27,28]. The stoichiometric HA has a monoclinic structure P2_1/_b, whereas in nature, due to the numerous substitutions with other ions, the hexagonal structure P6_3/_m appears [29]. FA has a hexagonal structure of P6_3/_m. Parameters of unit cells a and c are similar in both minerals and amount to a = 9.43, c = 6.88 for HA and, respectively, a = 9.37, c = 6.87 for FA [30,31]. Parameters a and c are slightly smaller for FA than HA, which results from the larger radius of the hydroxyl ion in relation to the fluoride ion (Figure 3). It is also observed that HA has lower crystallinity compared to FA.

In the interior of apatite crystals, there are channels with a diameter of 0.30–0.35 nanometers parallel to the c-axis. The inside of these channels is occupied by the ions located in the columns, hydroxyl groups for HA and fluoride ions for FA [32]. Studies by Laghizizil et al. [31] showed that FA is a better conductor than HA, which is due to the possibility of hopping fluoride ions in channels along the axis of the unit cell from the lattice sites in interstitial sites and back again [31]. In the stoichiometric FA, it is also possible to move the F^−^ ions into other positions by thermal activation, such as Schottky defects, characterized by high activation energy [31]. In the case of HA, proton conduction has a different mechanism. It can occur through conduction between adjacent hydroxyl groups OH^−^, OH^−^ (OH^−^ + OH^−^ → O^2−^ + HOH) or as a result of the proton jump from one OH^−^ group to the next via the PO_4_^3−^ ion. It is worth noting that the second mechanism takes place in the course of tooth decay by protonating PO_4_^3−^ ions. Probably, proton transport along the OH group chain is the first stage of the acid attack (beginning with tooth demineralization), leading to carious lesions [31,32]. The HA structure also indicates the second mechanism: The distance between OH^−^ groups is 3.44 Å, which is too large for the proton to jump, while the distance between OH^-^ and PO_4_^3−^ is 3.07 Å and is suitable for hydrogen bonding [31,32].

Physicochemical studies of FA compared to HA powdered samples indicate a higher FA density. Investigations into HFA samples with different fluoride ions and hydroxyl groups indicate an increase in density with an increase in fluoride ions [27,28]. However, the surface area of FA samples presents lower values than for similarly obtained HA samples [27,28].

Hardness, elastic modulus and fracture toughness for HFA samples with different proportions of F^−^ to OH^−^ ions were also tested. The highest hardness and flexibility were observed for the FA sample, but this value grows linearly with the increase in the ratio of F^−^ to OH^−^ ions, only in the case of flexibility. The highest resistance to cracking had a sample with an F/OH ratio of 0.6 [33]. Based on these results, Gross et al. recommended using materials with a lower content of F^−^ ions [33]. The HFA materials reveal much higher thermal stability compared to pure HA and FA [28].

During the biocompatibility tests for HA, FA and HFA, a significant difference in the dissolution of minerals was noticed. The ion release from HA, FA and HFA with different F^−^ ion ratios to pure water and cell cultures indicated a decrease in solubility with an increase in the content of fluoride [34,35,36]. Tredwin et al. claimed that the addition of F^−^ ions allowed the control of the dissolution rate of the obtained materials.

Biological tests also indicate better biological properties of FA or fluorhydroxyapatite (FHA) than HA [35,36]. Better cell proliferation, together with a smaller number of dead cells, was observed on the surface of FA and HFA [34,36].

## 2. The Role of HA and FA in Conservative Dentistry

Conservative dentistry focuses on keeping natural teeth in a healthy state. It includes prophylactic treatments aimed at restoring the natural appearance of teeth (whitening) or strengthening the dental tissue, as well as getting rid of changes associated with certain diseases (e.g., dental caries, tooth hypersensitivity). In the case of conservative dentistry, we usually deal with the use of gels, toothpastes and mouthwash.

### Toothpastes with the Addition of HA or FA

In toothpastes, particularly noteworthy is HA in the form of nanocrystals (nHA), because, in this form, it is easier to dissolve [37,38]. It is worth mentioning that tooth enamel can be gradually demineralized due to various mechanisms, among which abrasion, attrition, abfraction and erosion can be distinguished. Abrasion is physical wear caused by exogenous material forced over tooth (e.g., excessive tooth brushing, presence of abrasives in toothpastes). Attrition and abfraction are also physical wear, but not connected to foreign objects. Attrition occurs as a result of the action of antagonistic teeth and abfraction is caused due to tensile or shear stress in the cemento-enamel region provoking microfractures in enamel and dentine. By contrast, erosion is chemical wear, which occurs as a result of acids acting on plaque-free tooth surface. Clinical and experimental observations show that aforementioned mechanisms rarely act alone and usually interact with each other [39]. Long-untreated enamel mineral loss can lead to dangerous exposure of dentin and even dental pulp and is associated with negative effects such as tooth sensitivity [38]. In order to prevent this threat, fluoride-containing pastes are often used. In 2006, in Europe, the first toothpaste containing nHA, an alternative to a fluoride paste, was introduced to help the remineralization and repair of enamel [40]. In the study that compared the effects of the above toothpastes (more specifically, toothpastes containing ZnCO_3_/nHA or nHA) and ammonium fluoride-containing toothpastes, it was proven that different toothpastes containing nHA had similar remineralizing properties for dentin and enamel and higher levels than fluoride-containing toothpaste [40].

**Figure 4:**
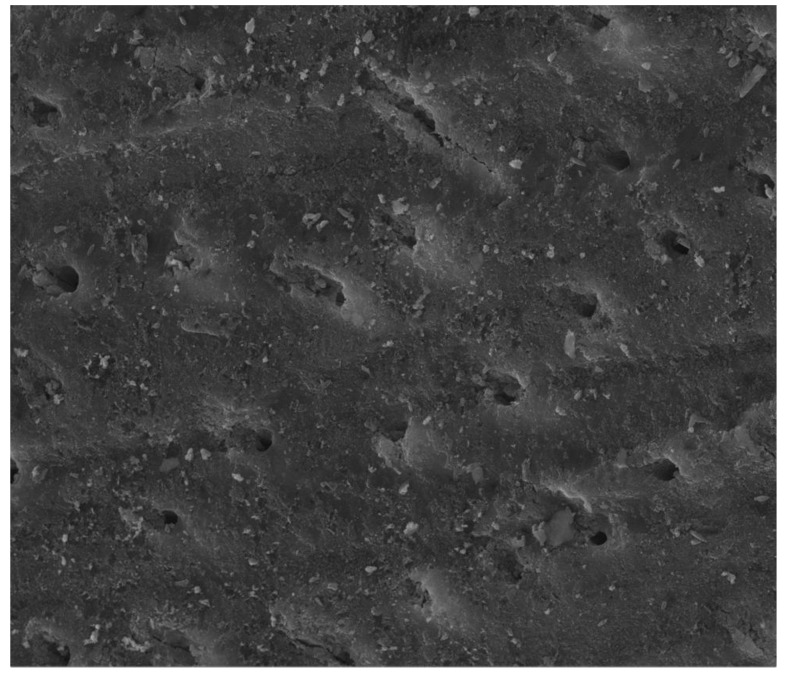
Scanning electron microscopy (SEM) micrograph of a dentin disc, showing notable tubule occlusion after keeping it in a mixture of artificial saliva and HA containing toothpaste for one week (6000× magnification) (reprinted from ref. [41] with permission).

In other studies, it was confirmed that the use of a toothpaste containing 2 wt.% of nHA reduced dental hypersensitivity in patients, which is related to the restoration of the enamel layer as a result of the remineralization and closure of the dentinal tubules [38,41,42,43]. Similar results were obtained in other studies [44,45,46], which explains why toothpastes containing nHA should be considered as among the first products used in the treatment of dental hypersensitivity and enamel restoration (including fluoride or calcium sodium phosphosilicates-containing toothpastes). nHA-containing toothpastes can also be used in the treatment of dental hypersensitivity after teeth whitening. This is confirmed by a study whose participants used a hydrogen peroxide gel for 14 days, after which they immediately washed their teeth with a toothpaste containing nHA. In the group of patients who did not use the nHA toothpaste, hypersensitivity appeared in more people and persisted for a greater number of days [40]. Other studies have confirmed the remineralization effectiveness of pastes containing HA and sodium fluorophosphate and a decrease in the demineralization process caused by the presence of an acidic environment [47]. In studies focusing on the determination of the effectiveness of the remineralization process and the closure of dentinal tubules as a result of using different toothpastes (fluoride toothpaste, HA or bioactive glass), it was found that the toothpaste with the best efficiency in closing the dentinal tubules was a toothpaste containing HA (Figure 4); in turn, fluoride toothpaste provided the best remineralization and increase in pH [41].

On the other hand, there are also studies that do not confirm the efficacy of using nHA-containing paste in the aforementioned conditions [48] or whose results are ambiguous [42]. In a study conducted by Hill et al. [42], dentin discs, after being treated with citric acid, were subjected to the use of dentifrices. In the SEM pictures, it was shown that toothpastes containing nHA and HA and doped with zinc (Zn-HA) significantly covered the dentin surface and closed the dentinal tubules, which was not observed in SEM images in the case of trials with toothpastes containing potassium oxalate or arginine and polyvinylmethyl ether/maleic acid (PVM/MA) copolymers. However, in the case of fluid flow reduction, the highest value was obtained for the dentin discs treated with toothpastes containing arginine and PVM/MA. The different results of these two tests may be explained by the fact that SEM microscopy shows only the surface of the dentin discs, while the state of deeper channels is not visible. In the available literature there are few papers on the potential use of FA or FHA in the treatment of hypersensitivity. Noteworthy is the study of Taha et al. [49] in which the toothpastes containing nano- and microcrystalline FHA were investigated. It turned out that the obtained pastes did not show cytotoxicity. Moreover, calcium, phosphorus and fluorine ions were released to provide sufficient occlusion of the dentin tubules.

In addition to the above uses, HA toothpastes have a positive effect on teeth whitening and brightness [50,51]. HA can also offer a gently smooth tooth surface [52]. Toothpastes containing zinc-doped HA (Zn-HA) are also of note [53,54]. One of the conducted experiments showed greater efficacy in protecting the enamel from demineralization caused by the acidic environment in favor of the paste containing Zn-HA (compared to the fluoride paste) [53]. Moreover, the presence of zinc should positively affect the state of the gums, thereby reducing the risk of parodontosis.

Mouthwash solutions containing nHA and enriched with zinc are also present on the market. They have similar effects to toothpaste, while additionally preventing the proliferation of bacteria in the oral cavity [55,56]. Examples of such fluids include products issued under the BioRepair^®^ brand, the advantage of which is a lack of chlorhexidine causing tooth discoloration.

**Figure 5:**
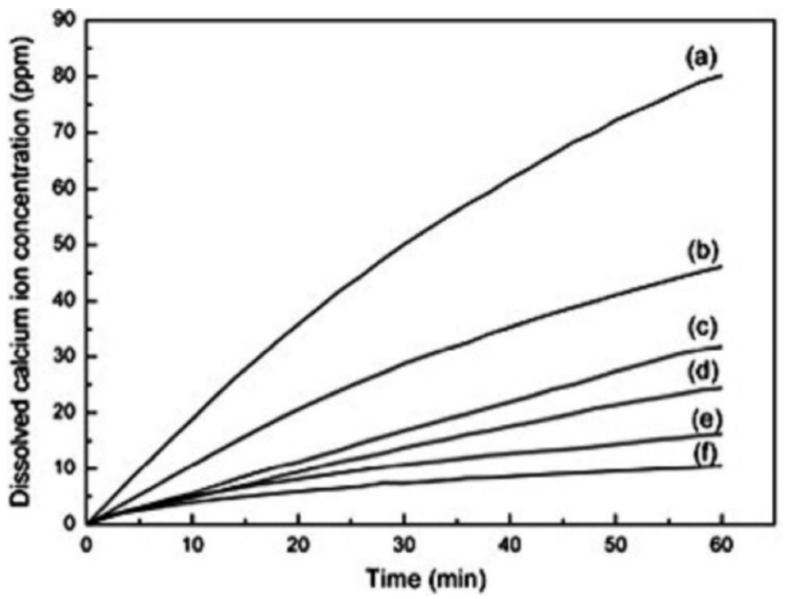
Dissolution behavior of powders with various amount of HA and FA: (**a**) 0% FA, (**b**) 20% FA, (**c**) 40% FA, (**d**) 60% FA, (**e**) 80% FA, (**f**) 100% FA in Tris buffer (pH 7.3) over a period of 1h (reprinted from ref. [62] with permission).

It should be noted that many toothpaste and mouthwash products contain fluorine. Consider that, in significant quantities, it is a toxic element. However, the use of small amounts of fluoride in these products results from its beneficial effects on the development of teeth and bones (fluorine stimulates osteoblast activity and thus accelerates bone regeneration). It protects tooth enamel from demineralization and thus caries [57,58,59]. It is also known that fluoride ions can substitute hydroxyl groups in enamel apatite, resulting in the formation of FA or FHA. As mentioned, this mineral is characterized by a greater resistance to acidic substances, lower solubility and greater hardness than HA (Figure 5), while retaining similar biocompatibility [21,24,59,60]. As a result, enamel is strengthened and erosion resistance is increased. In one study, the effectiveness of toothpaste containing FHA on enamel reconstruction was checked. The results established that FHA, inducing the growth of nanocrystals on natural enamel tissue, led to the formation of a synthetic layer of enamel. Therefore, this material, like FA, may be used, for example, in the treatment of cavities caused by caries [61].

Finally, it is worth presenting one more potential use of nHA, which is associated with the increasing occurrence of the problem concerning the erosion of enamel, especially in children and adolescents, which is caused by the growing amount of acidic foods and drinks in their diet (e.g., high popularity of energy drinks in recent years). One of the investigations focused on the effect of the Powerade^®^ drink on enamel erosion, to which nHA was added in various concentrations. The results showed that microhardness increased on the tooth surface as the concentration of nHA increased, which indicated a decrease in the negative effect of the beverage on the enamel [40]. Similar results were obtained in another study, where, after exposure to beer in order to demineralize the enamel, teeth were then soaked in a solution containing nHA. The measured microhardness of the enamel surface in this case was slightly smaller than the initial microhardness (before soaking the teeth in beer), while, in the case of a control (where the teeth were not exposed to the nHA solution after steeping in beer), the difference in microhardness was statistically significant [40]. The same effect can be observed with other beverages containing H_2_CO_3_, such as Coca-Cola^®^ [53].

## 3. Dental Implantology

In the case where prophylaxis or non-invasive treatment of dental diseases is insufficient, invasive methods are often necessary. In this case, HA is used as the implant material or the component of such a material, which, for example, can be in the form of implant coatings, scaffolds, blocks and cements.

### 3.1. Hydroxyapatite Coatings

**Figure 6:**
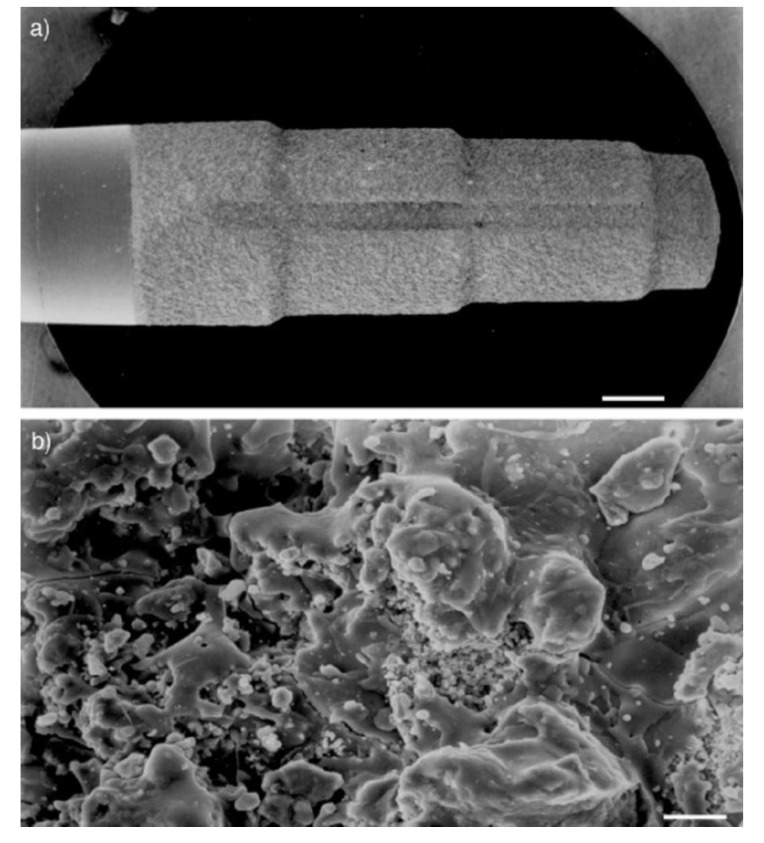
Dental implants (by Friadent) coated with CaP by a plasma-spray process (**a**: 10×, **b**: 1000× magnification; **a**: 1mm, **b**: 10 µm scale bars) (reprinted from ref. [69] with permission).

In recent times, due to the ageing of the world’s population, tooth loss is becoming a growing public health problem. On the one hand, it leads to difficulties in taking food through the disruption of mastication, while, on the other, it increases the risk of damage to the bone and surrounding tissues. Often, a dental implant is sometimes the most effective solution allowing the reconstruction of the dental arch [63]. As a result, a significant development in therapies using tooth implants can be noticed in the last 40 years. During this period, researchers have focused on obtaining materials with appropriate mechanical properties and characterized by biocompatibility in relation to human tissues. Currently, the most commonly used are titanium implants, made of pure titanium or titanium alloy and other metals (e.g., Ti-6Al-4V) [63,64,65]. These materials are characterized by high mechanical strength and high corrosion resistance and show strong biocompatibility in relation to human tissues in vivo [64]. A pivotal factor is also the surface of such an implant, because it is the only element that comes into contact with human tissues, thus affecting the biological response of the body. In the case of a titanium implant, this is associated with a long treatment period, where there is no direct connection between the implant and the living bone. In order to overcome the above-mentioned unfavorable features, acid treatment of the surface of the titanium implant is often used to increase its roughness. This in turn affects the better attachment of bone cells to the implant, as well as increasing their differentiation and growth [66]. However, a more beneficial way to obtain the best biological response of the body is the use of coated implants, where one of the materials used is HA (Figure 6) [67,68,69,70]. 

In one of the conducted tests, the surface area of the titanium implant subjected to acid etching and the identical implant covered with HA coating were compared. The results showed that the coated implant had a 70% greater surface area compared to the uncoated implant [40]. Thus, it can be concluded that the coated implant will have a larger contact surface with bone tissue.

**Figure 7:**
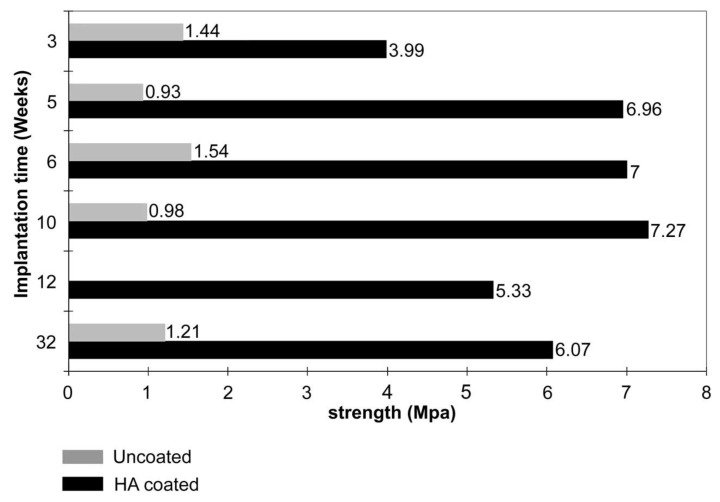
Shows improvement of the interfacial bond strength in porous titanium with plasma-sprayed HA coating in comparison with uncoated porous titanium (reprinted from ref. [76] with permission).

HA not only significantly improves the biocompatibility of the implant with bone tissue, but also has a positive effect on osseointegration (consisting of the creation of a specific binding between the implant and bone tissue surrounding the defect) and osteoconduction (induction of bone cell growth and development). As a consequence, this leads to faster regeneration of bone tissue in the area of the defect, thus reducing the risk of bacterial infection, provides better stability of the implant, and reduces the risk of its rejection by the body [65,70,71,72,73,74,75]. Clinical studies have shown that the lifetime of an HA-coated implant is much longer than that of an uncoated implant (Figure 7) [12,68,76]. In addition, HA can be used as a carrier of growth factors, which additionally positively affects the proliferation of bone tissue cells [9]. A study in which the impact of loads related to the functioning of the tooth (occlusal loading) on periodontal regeneration, using a titanium implant with HA coating was checked (the implant was introduced into a rat’s alveolus after molar extraction) is also noteworthy. In the case of an implant where there was no occlusal loading, there was a direct connection of the implant with the bone. In turn, in the test, where the implant was subjected to the above loads, there was a fibrous formation of the peri-implant tissue between the implant and the bone [77].

**Figure 8:**
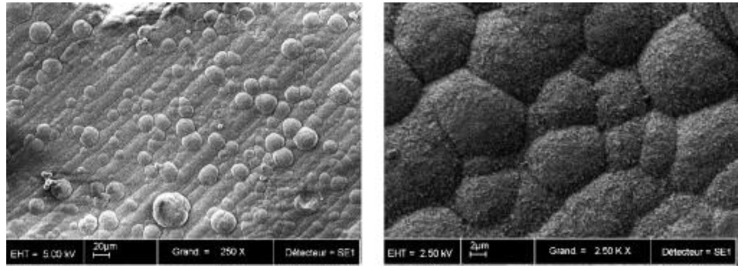
SEM micrographs of biomimetic calcium phosphate coating. Magnification: 250× (left) and 2500× (right) (reprinted from ref. [74] with permission).

There are several methods for coating a titanium implant with HA material, which can be divided into chemical and physical methods. The chemical methods include sol-gel, electrochemical deposition, biomimetic process, electrophoretic deposition, and micro-arc oxidation MAO. Physical methods include plasma-spraying deposition, physical vapor deposition, magnetron-sputtering deposition, ion beam-assisted deposition, pulsed laser deposition and hot isostatic pressing [52,64,65]. These methods have different advantages and disadvantages (e.g., physical methods allow for a stronger connection between the implant and the coating, but they make it difficult to coat geometrically complex surfaces; meanwhile, chemical methods are much more effective in such cases, enabling the incorporation of bioactive agents) [64]. Currently, the most widely used commercial coating technique for the implant is plasma spraying (Figure 8) [9,65,67,74,78,79]. Unfortunately, this method has several adverse effects, such as low resistance to cracking or a lack of uniformity of the coating. In addition, the coatings obtained in this way are usually characterized by a thickness of several dozen μm, which increases the risk of negative phenomena such as the cracking, decay or desorption of the coating. This results in a mixed opinion about the use of HA-coated titanium dental implants by plasma spraying; however, a systematic review did not reveal the reduced viability of such an implant in relation to other types of implants used in dentistry [79]. The previously mentioned significant thickness of the coating resulted in the initiation of research into techniques aimed at obtaining a coating thickness of only a few or a dozen microns, which would eliminate or reduce the risk of negative phenomena accompanying thicker coatings [71,79]. Some of the experiments carried out so far have promising results [75,78], while others have focused on the use of HA, not as a coating, but in the form of “islands” in the titanium matrix, which would have the same effect and remove the adverse effects of the coating, while, on the other hand, increasing the positive effect on the development of bone tissue [72,80]. Some studies reveal a similar effectiveness of coatings using bioactive glasses for HA coatings [81,82].

Finally, it is worth mentioning the potential use of FA as a dental implant coating. This is due to its poor solubility in the acid environment and stability, which extends the life of the implant material and enhances the osseointegrating properties of the implant with the bone tissue in relation to the HA coatings [83].

### 3.2. Hydroxyapatite Scaffolds and Blocks

As mentioned earlier, HA, due to its poor mechanical properties, can be used as an independent material but only in the case of the implantation of elements with low mechanical tension. Therefore, HA has beneficial properties in maxillofacial surgery, where it is often used with both smaller 3D shapes and larger blocks [12,84].

Among the above HA materials, we can distinguish highly porous and dense materials. Porous materials are characterized by higher osteoconductivity and faster resorption, while compact HA is characterized by higher mechanical properties [7]. One study examined the effect of various binders, such as polyvinyl alcohol (PVA), starch and cellulose, on the synthesis of porous HA shapes. The results showed that it was possible to obtain shapes with different porosities and densities, which were thus capable of carrying various tensions [85].

These materials are used in bone cavity restorations in maxillofacial surgery, which is especially important in the case of jawbone atrophy. Atrophy of the jawbone occurs most often as a result of missing teeth cavities after losing teeth (e.g., due to caries); another cause may be, for example, parodontosis [86]. In the case of the loss of the upper teeth, there is also a reduction of the maxillary sinus floor. As a consequence, functional disturbances in masticatory system occurs and, with time, pain and inflammation of the temporo-mandible joint, headaches or bite abnormalities may occur. In addition, in the case of the loss of the lower teeth, the corresponding upper teeth may slip out, which in turn will significantly hinder the subsequent insertion of the implant in the area of the defect [87,88]. To prevent this, alveolar ridge augmentation (controlled regeneration) and maxillary sinus floor elevation are used, where HA in the form of granules or blocks is used as a filling material. This leads to the reconstruction of the lost bone, which will be the base for dental implants [7,52,89]. A study in which a composite nHA/polyamide was fabricated in the form of a membrane for potential use in controlled bone regeneration is of relevance. A membrane with an asymmetrical pore distribution was obtained (on one side, there was a compact layer of micropores, while there was a spongy layer of macropores on the other). The micropores prevent the migration of fibrous connective tissue; at the same time, they allow the passage of ingredients involved in the regeneration of bone tissue. The resulting material showed strong affinity for binding bone marrow stromal cells and showed no negative effect on cell proliferation, thus indicating a good biocompatibility of the synthesized membrane and the possibility of its use in controlled bone regeneration [40].

It is also important to note that modified HA are used. An example of a product containing carbonated HA (CHA) is the bioactive resorbable OsteoGen^®^ material. It has a crystalline apatitic structure and also possesses strong hydrophilic properties, which allow the material to absorb liquids easily. It is intended for lifting the bottom of the sinus, as a filling after extraction of the tooth and for repairing periodontal bone defects [7].

### 3.3. Cements

CaPs cements belong to the group of self-hardening materials, which solidify after entering the target site [90]. They are made of one or more calcium orthophosphates powders, such as tetracalcium phosphate (TTCP), dicalcium phosphate (anhydrous DCPA or dihydrated DCPD), tricalcium phosphates (α or β form of TCP), HA etc. and a small amount of liquid (usually water), that upon mixing is leading to one of two end products: Brushite cement or apatite cement [9,91]. We limited this review to the CaPs cements, in which HA is used also as starting material. It should be noted that the main advantages of using HA in the form of cement are fast setting time, high biocompatibility and osteoconductivity, good plasticity, and easy delivery of material to the target site [92].

Cements can be used to cover the pulp of permanent teeth and as a filling of root canals [91]. They are also used as sealing materials for the dental canal, where the first documented application of calcium-phosphate cement in this way dates back to 1985 [93]. Very interesting studies have been conducted following the extraction of the premolars of the mandible in Beagle dogs and the removal of part of the alveolar bone, where a block of HA was introduced into the defect, previously prepared from HA cement. The results showed that, a month later, the implantation of the new bone took place, which over time replaced the HA block introduced in place of the defect [94]. In the case of cranio-facial or maxillo-facial surgery, HA cements are readily used materials, firstly because of their ease of application, and secondly because of the plastic properties that significantly improve the cosmetic and aesthetic fit of the implant. They are used, for example, during the regeneration of facial bones within the sinuses or for the capping of holes after neurosurgical procedures [91]. Studies on animals and humans have confirmed the effectiveness of using HA cements [95,96]. Furthermore, due to the low temperature of freezing and injectability, HA cements may be good candidates for drug carriers or biologically active ingredients. Adding them to the liquid phase would allow for the even distribution of the cement. Examples are growth factors that cannot be injected on their own, because they would not induce tissue formation and regeneration due to their rapid migration from the implant site after injection. The use of HA cement as their carrier would facilitate the controlled release of these factors to the level at which the therapeutic effect would be obtained. In addition, due to the use of low concentrations of growth factors in bone tissue engineering, their effect on the cementation of the cement would be insignificant [63].

Studies were also conducted with glass ionomer cements (GICs) or composite resins, to which HA was added. These are popular materials used as fillings in dental cavities, while the above-mentioned studies show that the addition of HA increases the strength of the binding of such cement and its physical properties [40,97,98,99]. Similar results were obtained in an experiment where HA was added to light-cured GIC (LC GIC). This is important because LC GIC shows a higher strength and drying rate and resistance to dehydration compared with traditional GIC, while HA also improves its properties [97]. The increase in cement-binding strength improves the maintenance of prosthetic restoration and reduces the possibility of secondary caries, which may lead to prolonged durability of the applied filling [97].

Similar studies were performed using FA. The higher hardness and lower solubility of this material allowed us to improve the mechanical properties of the GIC compared to HA [100,101,102], and also ensure better stability of the material in vivo [62]. Finally, it is worth mentioning another study involving FA, in which, due to the poor antibacterial activity of fluoride, the above material was doped with silver ions. As silver ions have a documented antibacterial effect, introducing them into the FA structure will improve the properties of the material. In studies, FA with a low content of silver ions was obtained, which revealed the antibacterial activity against *K. pneumonia*, *S. aureus* and *M. luteus* bacteria [59].

## 4. Summary

Over the years, HA has prompted increasing interest among researchers due to its unique properties. With the development of science, the use of this mineral has increased in further branches of science, including dentistry. Due to its similar structure to human bone tissue and biological properties, HA (and its derivatives, e.g., FA) is today one of the most popular materials used in dental implantology, as well as in the treatment and prophylaxis of dental diseases. This mineral is readily used in combination with other materials, which allows for an overall improvement in the properties of the product obtained and the marginalization of the defects of individual components. HA material is one of the most promising compounds used in dentistry, which is confirmed by the growing amount of research and work devoted to the development of this material.
